# Functional and Structural Changes of the Blood-Nerve-Barrier in Diabetic Neuropathy

**DOI:** 10.3389/fnins.2018.01038

**Published:** 2019-01-14

**Authors:** Mette Richner, Nelson Ferreira, Anete Dudele, Troels S. Jensen, Christian B. Vaegter, Nádia P. Gonçalves

**Affiliations:** ^1^Danish Research Institute of Translational Neuroscience, Nordic EMBL Partnership for Molecular Medicine, Department of Biomedicine, Aarhus University, Aarhus, Denmark; ^2^The International Diabetic Neuropathy Consortium, Aarhus University Hospital, Aarhus, Denmark; ^3^Center of Functionally Integrative Neuroscience, Institute of Clinical Medicine, Aarhus University Hospital, Aarhus, Denmark; ^4^Department of Neurology, Danish Pain Research Center, Aarhus University, Aarhus, Denmark

**Keywords:** blood-nerve barrier, diabetic neuropathy, peripheral nerve, diabetes, microvascular liabilities

## Abstract

The incidence of diabetes mellitus is approaching global epidemic proportions and should be considered a major health-care problem of modern societies in the twenty-first century. Diabetic neuropathy is a common chronic complication of diabetes and, although an adequate glycemic control can reduce the frequency of diabetic neuropathy in type 1 diabetes, the majority of type 2 diabetic patients will develop this complication. The underlying cellular and molecular mechanisms are still poorly understood, preventing the development of effective treatment strategies. However, accumulating evidence suggests that breakdown of the blood-nerve barrier (BNB) plays a pivotal pathophysiological role in diabetic neuropathy. In the present review, we highlight the structural and functional significance of the BNB in health and disease, focusing on the pathological molecular events leading to BNB dysfunction in diabetic neuropathy. In addition, we discuss potential molecular targets involved in BNB homeostasis that may pave the way toward novel therapeutic strategies for treating diabetic neuropathy.

## Introduction

The world is currently experiencing an unprecedented increase of diabetes mellitus, globally affecting 415 million people and with numbers increasing exponentially predominantly due to type 2 diabetes (T2D), expecting to reach 642 million people by 2040. Furthermore, prediabetes, which constitutes a state of high-risk for T2D development with a yearly conversion rate of 5–10%, is predicted to reach a prevalence of 471 million people globally by 2035 (Bansal, 2015). Diabetic neuropathy (DN) is the most common diabetic complication characterized by damage to nerve glial cells, their axons and endothelial cells, with a prevalence ranging from 30% up to 50% of T2D patients (Peltier et al., 2014). In over 50% of DN patients, substantial, irreparable nerve damage occurs prior to diagnosis, making this condition the leading cause of diabetes-related hospital admissions and non-traumatic amputations in the Western world. Despite these serious complications, development of targeted therapies for DN has been hindered by the lack of understanding of the complexity and different etiologies of this disorder. Nevertheless, a number of mechanisms have been proposed ranging from increased oxidative stress, mitochondrial dysfunction, inflammation and protein glycosylation (metabolic hypothesis) (Feldman et al., 2017; Hinder et al., 2018) to neurovascular disturbances with microangiopathy ultimately reducing the amount of oxygen and glucose that can be extracted by the nerve cells (vascular hypothesis) (Cameron et al., 2001; Tesfaye and Selvarajah, 2012). Both hypotheses are valid, not exclusive and most likely interacting for the development and progression of DN.

Most clinical and basic research has been focused on diabetic axonopathy; however, increased awareness of molecular alterations in Schwann cells (SCs) is now emerging, highlighting the importance of schwannopathy for the development and progression of DN (Goncalves et al., 2017). Impaired SC function, previously demonstrated by the loss of axonal associations, reduced expression of myelin-associated proteins (Kawashima et al., 2007) and neurotrophic factors (Calcutt et al., 2003; Dey et al., 2013), might also affect vascular and perineurial components of the blood-nerve interface by altering endothelial cell function (Chapouly et al., 2016). Endoneurial capillary dysfunction with increased thickness of the basement membrane, loss of pericytes and endothelial hyperplasia are diabetes-induced microvascular liabilities often found in nerve biopsy samples from patients with DN (Giannini and Dyck, 1995), overall suggesting compromised blood-nerve barrier (BNB) function. In contrast to the blood-brain barrier (BBB), limited information is available regarding BNB biology and pathological mechanisms activated upon different disease states affecting the peripheral nerves, such as DN. Here, we review basic concepts of BNB anatomy and function and discuss how microvessel dysfunction is believed to contribute to the pathogenesis of DN, aiming at identifying future targets for therapy and new important lines of research.

## Barriers of the Nervous System: an Overview

A defined group of highly selective semipermeable barriers controls the exchange between the blood and the nerve tissue by limiting passive diffusion of blood-borne solutes while actively transporting mandatory molecules. The BBB and the BNB play key roles in maintaining the integrity of the respective compartments of the nervous system, and increasing evidence suggests that their breakdown drives the initial pathogenic events leading to a multitude of inflammatory or immune-mediated neuropathies or neurodegenerative diseases (Nelson et al., 2016; Sweeney et al., 2018).

The BBB is composed of a confluent layer of highly specialized microvascular endothelial cells covering the capillaries intertwined by astrocytic processes forming tight junctions (TJs) in the brain. In addition, brain capillaries are shielded by mature pericytes sharing the basement membrane with the endothelium, which together with astrocytes secret basement membrane matrix proteins (Reinhold and Rittner, 2017). Altogether, the BBB is by far the largest interface for molecular blood–brain exchange and in the adult human comprises an estimated surface area between 12 and 18 m^2^ (Nag and Begley, 2005), meaning that no brain cell is farther than approximately 25 μm away from a capillary. Thus, cells within the CNS are freely accessible to circulating macromolecules, proteins and drugs, provided that these can cross the BBB (Abbott et al., 2010). While the BBB is fairly permeable to small molecules and lipid-soluble proteins, larger molecules require receptor-mediated transcytosis to enter the central nervous system (CNS) (Yarlagadda et al., 2007).

**FIGURE 1:**
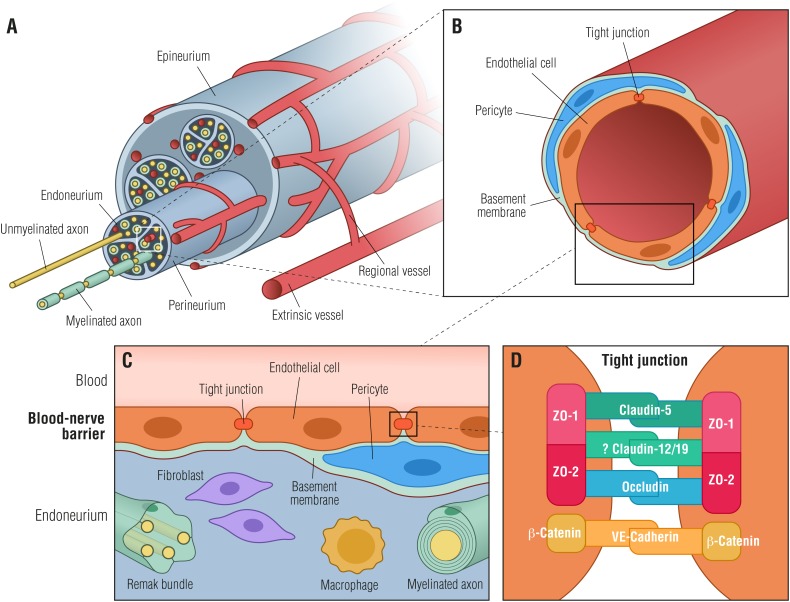
Blood-nerve barrier. **(A)** Transverse view of a peripheral nerve ensheathed by epineurial collagen fibrils (epineurium) and blood vessels. Individual nerve fascicles consisting of unmyelinated and myelinated axons as well as small blood vessels are ensheathed by the perineurium, forming the endoneurial microenvironment. **(B)** Individual endoneurial blood vessel surrounded by endothelial cells, pericytes and the basement membrane. **(C)** Cellular structure of the blood-nerve barrier, formed by endothelial cells, that are connected by tight junctions, pericytes and the basement membrane. The barrier is exposed to cells and molecules circulating in the blood, protecting constituents of the endoneurium (Remak bundles, myelinated axons, resident macrophages and fibroblasts) from toxic factors. **(D)** Endothelial cells are tightly interconnected by tight junctions and adherens junctions forming a restrictive intercellular barrier. Zona occludens-1 and -2 (ZO-1, ZO-2) interact with claudin-5, occludin and likely with claudin-12/19 forming tight junctions. β-catenin forms in conjunction with VE-cadherin adherens junctions.

The BNB shares many structural features with the BBB (Figures 1B,C) with the exception of astrocytes and the glial limiting membrane formed by astrocytes, defining the dynamic but selective blood-tissue interphase within the peripheral nerve endoneurium. Thus, in order to reach the endoneurial extracellular space, blood-borne molecules need to cross the endoneurial vascular endothelium or the perineurium that surrounds the nerve fascicle (Figure 1A).

An essential component of the BNB cellular architecture in both perineurial and endothelial cells is the TJs (Figures 1B–D). The TJs consist of a complex network of transmembrane and peripheral proteins such as claudins, occludins, junctional adhesion molecules, and zonula occludens (ZO) complexes that are important for restricting the paracellular flow of ions and molecules into the endoneurial milieu (Runkle and Mu, 2013). Of particular relevance are claudins, of which claudin-5, -3, and -12 are highly expressed in the BBB (Nitta et al., 2003; Wolburg et al., 2003; Lal-Nag and Morin, 2009), whereas claudin-1 is located in the adult human perineurium (Pummi et al., 2004). Although most studies on the function of claudins have been focusing on the BBB, it has been shown that secretion of bFGF by BNB-derived pericytes strengthens the barrier function by increasing the expression of claudin-5 (Shimizu et al., 2011b). Taking into account that the BNB does not contain cells equivalent to astrocytes, which control the BBB functions, peripheral nerve pericytes may be the main regulators of the BNB basement membrane (Shimizu et al., 2011b). Very recently, a pioneer study using RNA-sequencing on cultured adult human primary endoneurial endothelial cells has shed some light on the human BNB molecular composition (Palladino et al., 2017). The authors identified transcripts defined as the normal adult BNB transcriptome including: (i) several transporters, (ii) chemokines and chemokine receptors, and (iii) immunoglobulin transporters or receptors such as FCGRT (Fc fragment of IgG receptor and transporter), which facilitates endothelial transcytosis of IgG (Palladino et al., 2017). Nevertheless, the knowledge on the molecular composition of the BNB remains mostly unknown and, as a consequence, the molecular changes occurring in the human BNB during development, in healthy homeostatic states as well as in pathologic conditions like DN, remain elusive.

## The Diseased Blood-Nerve Barrier

Several forms of neuronal damage can alter the delicate BNB balance, by locally or systemically increasing BNB permeability, ultimately leading to neuronal dysfunction and contributing to the development of neuropathy. Pathological BNB breakdown involves two key features: (i) paracellular leakage of potentially harmful molecules into the nerve tissue and (ii) upregulation of adhesion molecules on BNB structures, allowing transcellular entry of hematogenous immune cells to the endoneurium where they engage in the local inflammatory cascade. But what initiates these pathological changes of the BNB?

### Local Nerve Injury

A local and somewhat limited response of the BNB may be elicited upon an externally induced nerve damage. Following a traumatic peripheral nerve injury, such as partial ligation of the sciatic nerve, nerve edema evolves in the distal segment caused by an increase in endoneurial fluid pressure accompanied by a long-lasting disruption of the BNB (Richner et al., 2011; Lim et al., 2014). It is well known that macrophages are important immune cells involved in the activation of inflammatory responses upon nerve injury and are an abundant source of cytokines. At the injury site, resident macrophages have been shown to alter the permeability of microvessels and to be important for the breakdown of the BNB via expression of the cytokine vascular endothelial growth factor (VEGF) (Shimizu et al., 2011a). This has been substantiated by intraneural BNB-bypassing injection of VEGF or serum collected from nerve injured mice, containing increased levels of cytokines (Wells et al., 1992; Lim et al., 2014), into naïve animals, which was found to increase BNB permeability and to induce mechanical allodynia (Lim et al., 2014). Increased BNB permeability appears to involve TJ changes allowing paracellular influx of molecules into the peripheral nervous system (PNS), as traumatic peripheral nerve injury was found to reduce the expression of ZO-1 in endoneurial vessels and its expression in the perineurium to be altered within injured fibers (Lim et al., 2014). Similarly in a rat model, loss of ZO-1 expression was found to be a key step in the breakdown of the BBB by altering cell-cell adhesion in involved blood vessels (Bolton et al., 1998), substantiating the importance of ZO-1 on TJ integrity in the blood-nerve interface.

### Diabetic Neuropathy

#### Increased BNB Permeability

The first reports connecting DN to altered BNB permeability appeared more than 30 years ago when studies on diabetic patients and rodent models of type 1 diabetes showed augmented endoneurial concentrations of large molecules like albumin, IgG, and IgM as well as increased flux of mannitol, reflecting increased BNB permeability (Rechthand et al., 1987). Elevated endoneurial concentrations of these molecules were also found in diabetic patients without neuropathy suggesting that increased BNB permeability may precede and contribute to the development of DN (Poduslo et al., 1988), which is substantiated by the finding that accumulation of proteins in the endoneurium can lead to dysregulation of the osmotic balance and potentially to development of nerve edema (Mizisin and Weerasuriya, 2011). However, studies on diabetic rats failed to show increased BNB permeability to large molecules (Sima and Hay, 1981), which might be explained by an increased BNB permeability to glycated albumin (Inaba et al., 2007). Increased permeability of BNB to glycated albumin has also been observed in healthy rats and, thus, elevated levels of non-enzymatically glycated proteins, and not BNB breakdown, might lead to protein accumulation in the endoneurial space in diabetes (Poduslo and Curran, 1992). Metabolic alterations, such as excess glucose and increased fatty acid flux, are well-known hallmarks of diabetes that, perhaps not surprisingly, may damage the BNB due to the continuous exposure of endoneurial endothelial cells to blood and to toxic circulating factors.

Albeit systemic breakdown of the BNB today is considered an initial key step in development of DN (Cameron et al., 2001), the pathology and pathogenesis remain unclear.

#### Neuroinflammation

Local nerve inflammation due to an externally induced nerve damage involves secretion of cytokines and chemokines by Schwann cells, which attracts leukocytes across concentration gradients, facilitating their extravasation at sites of injury (Ydens et al., 2013). Locally increased BNB permeability may therefore be an immediate inflammation-mediated response (Harvey et al., 1995; Moreau et al., 2016), that, similarly to acute inflammation, may last a few hours to a few days, upon which tissue normality is restored.

However, in some cases, inflammation and aberrant BNB permeability appear uncontrollable, contributing to the development and progression of neurodegenerative disorders like DN. DN is not usually categorized as an immune-mediated neuropathy, however, microvascular changes induced by the inflammatory cascades, as reviewed in Nguyen et al. (2012), are observed in at least some patients. Likewise, genes associated with inflammation and immune regulation have been found upregulated in mouse models of T2D, suggesting that dysregulated immune-related processes and cell infiltration may have a contributory role in DN onset (O’Brien et al., 2015; Hinder et al., 2018). Involvement of macrophages in DN is supported by the increased expression of genes related with phagosome formation in the nerve tissue and by alterations in pathways associated with Toll-like receptor (TLR) signaling (Zhu et al., 2015; Hinder et al., 2018). Macrophages have in addition to BNB breakdown after traumatic nerve injury (Shimizu et al., 2011a), been associated with spontaneous resolution of transient inflammatory pain (Willemen et al., 2014). It may therefore be speculated that BNB breakdown causes entrance of cells and molecules from the blood to the endoneurium to an extent that cannot be counteracted by the pain resolving abilities of anti-inflammatory macrophages, thereby contributing to pain development associated with diabetic inflammation. Chronic inflammation is often coupled with microvascular disturbances in diabetes, as inflammatory mediators can augment the expression of thrombosis-related genes in endothelial cells, thereby amplifying the inflammatory response (Grant, 2007). Recently, a study on human diabetic sural nerve biopsies found that augmented levels of CD40, a surface protein of e.g., macrophages, mediated the interactions between inflammation and thrombosis, emphasizing the contribution of microangiopathy to nerve fiber loss in DN (Kan et al., 2018).

#### Endoneurial Hypoxia

**FIGURE 2:**
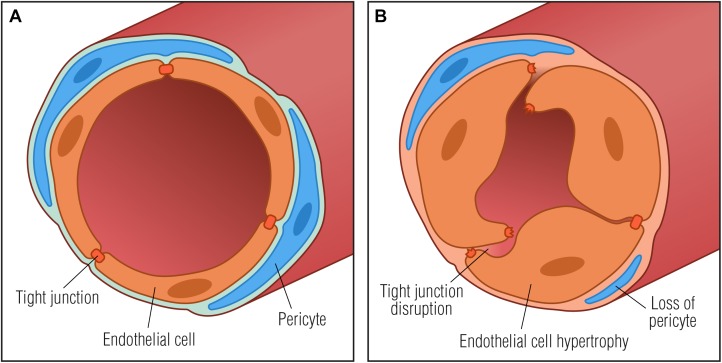
Basement membrane thickening in diabetic neuropathy. **(A)** Normal vessel with endothelial cells connected by tight junctions and embedded in a basement membrane (light blue) with surrounding pericytes, forming a restrictive barrier between the blood and the endoneurium. **(B)** Pathological vessel abnormalities that may further contribute to diabetic neuropathy progression: thickening of the basement membrane, degradation of tight junctions and endothelial cell hypertrophy that may result in microcirculatory disturbances in the endoneurial space by compromising capillary luminal area.

Microvascular abnormalities in the endoneurium that reflect increased BNB permeability are degradation of paracellular TJs, loss of microvascular pericytes, increased basement membrane thickness, increased proportion of fibrin positive blood vessels as well as endothelial cell hyperplasia (Figure 2; Malik et al., 1989, 2005; Giannini and Dyck, 1995; Cameron et al., 2001; Thrainsdottir et al., 2003; Mohseni et al., 2017; Kan et al., 2018). Basement membrane thickening around endoneurial microvessels is the most notable pathological abnormality in DN and may result in microcirculatory disturbances in the endoneurium leading to tissue hypoxia and further worsening of peripheral neuropathy. Diabetic endoneurial hypoxia has been corroborated by the finding of reduced oxygen tension (Tuck et al., 1984; Newrick et al., 1986; Cameron et al., 1994; Ibrahim et al., 1999) and elevated expression of HIF-1α (Kan et al., 2018) in nerves from diabetic patients, further highlighting involvement of microangiopathy in the pathology of diabetes. Endoneurial hypoxia of functional origin has also been proposed (Ostergaard et al., 2015), however, it remains to be assessed experimentally. Interestingly, in a non-diabetic mouse model of peripheral nerve injury, endoneurial hypoxia has also been demonstrated together with the development of endoneurial vessel basement membrane thickening and endothelial cell hypertrophy. In a mouse model of externally induced nerve injury, it has been suggested that hypoxia drives opening of the BNB through the VEGF pathway (Lim et al., 2015). Since hypoxia is known to induce VEGF upregulation in macrophages (Xiong et al., 1998), and VEGF has been found to alter the permeability of microvessels and to be implicated in the breakdown of the BNB (Shimizu et al., 2011a; Lim et al., 2015), it is tempting to speculate if diabetic hypoxia *per se* may induce upregulation of VEGF in resident or systemic macrophages thereby contributing to increased BNB permeability.

Under conditions of hypoxia, the transcription factor hypoxia inducible factor-1 (HIF-1) has been found to accumulate and activate hypoxic cellular transduction pathways normally inducing tissue repair (Lokmic et al., 2012). However, in a diabetic mouse model of ischemic stroke, HIF-1 upregulation, along with VEGF upregulation, has been found to be deleterious, contributing to the disruption of the BBB (Zhang et al., 2016). In diabetic patients, augmented levels of neuronal HIF-1 has recently been found to be associated with increased CD40 expression in endoneurial capillaries, reflecting increased macrophage and T-cell infiltration. Additionally, HIF-1 upregulation was important for phosphatase and tensin homolog (PTEN) overexpression, which has been found to contribute to impaired regeneration of diabetic axons (Park et al., 2010; Singh et al., 2014), and for all molecules correlated with the morphometric index of vascular integrity (Kan et al., 2018). HIF-1α derived from low oxygen tension can also lead to an increase of NADPH oxidase 2, which is a major source of tissue damaging reactive oxygen species (ROS) in vessel walls (Yuan et al., 2011; Goncalves et al., 2017). On the other hand, ROS is a key mediator of oxidative damage, tight junction modification and matrix metalloproteinases activation leading to BBB breakdown in the central nervous system (Pun et al., 2009). Similarly, in diabetic neuropathy, ROS can lead to vascular endothelium dysfunction with reduced nerve perfusion and endoneurial hypoxia, resulting in conduction deficits (Cameron and Cotter, 1999). Endoneurial hypoxia can then activate other molecular pathways as inflammation, culminating with a vicious cycle of oxidative and nitrosative stress adding to the breakdown of the BNB and ultimately exacerbating neuropathy.

#### Pericyte Dysfunction

Studies on human nerve biopsies have shown degeneration of endoneurial microvessel pericytes in diabetes (Giannini and Dyck, 1995). Pericytes are mural cells embedded directly in the basement membrane surrounding endoneurial capillaries and can play a role in maintenance of endothelial cell barrier properties (Armulik et al., 2005; Figure 1B). Pericytes have the potential to act as paracrine cells for endothelial cells, as pericyte cell lines derived from human peripheral nerves have been found to express a number of soluble growth factors (e.g., Ang1, VEGF, TGF-β, and bFGF), which are important for regulation and maintenance of the BNB (Shimizu et al., 2011a). Incubation of peripheral nerve microvascular endothelial cells with medium from peripheral nerve pericytes increased endothelial cell expression of the TJ protein claudin-5 (Shimizu et al., 2011a; Figure 1D), suggesting a role of pericytes in regulation of BNB permeability. When pericytes are lost, as it has been occasionally observed under diabetic conditions (Cameron et al., 2001), their paracrine BNB regulation is absent or significantly reduced, contributing to BNB breakdown (Giannini and Dyck, 1995). Furthermore, the diabetic environment has also been found to affect pericyte production of fibronectin and collagen type IV, two important extracellular matrix components of the BNB basement membrane (Shimizu et al., 2011b). Likewise, pericyte production of fibronectin and collagen type IV has been shown to be upregulated after *in vitro* exposure to elevated levels of advanced glycation end-products (AGE), which are late products of non-enzymatic glycation known to be increased in diabetes as a consequence of hyperglycemia and/or dyslipidemia (Shimizu et al., 2011b; Rhee and Kim, 2018). This effect may, at least partially, explain the basement membrane hypertrophy often observed in endoneurial capillaries of subjects with DN (Giannini and Dyck, 1995; Shimizu et al., 2011b). In addition, exposure to AGEs have been shown to increase pericyte production of VEGF and TGF-β that in turn can either stimulate production of fibronectin and collagen type IV or exert a paracrine effect on endothelial cells (Shimizu et al., 2011a,b). The interplay between microvascular endothelial cells and pericytes has further been substantiated by the finding that endothelial cell exposure to AGEs decreased production of claudin-5 via increased VEGF (Shimizu et al., 2011a). These effects can potentially contribute to dysregulation of the BNB in diabetes and to basement membrane hypertrophy. It has also been suggested that pericytes can sense hypoxia in the CNS, and potentially in the PNS (Armulik et al., 2005). If so, pericytes can respond to endoneurial hypoxia by altering some BNB properties.

Altogether, loss of pericytes in DN can potentially lead to disintegration of the BNB, similarly to the process in diabetic retinopathy where pericyte loss from retinal microvessels is one of the first observed cellular deficiencies (Armulik et al., 2005). However, it is important to note that not all the studies have found pericyte loss in DN and therefore their role for neuropathy progression currently remains unclear (Malik et al., 2005).

## New Cellular Tool for BNB Modeling

Despite structural differences, BNB pathophysiology appears to share similarities with BBB alterations upon traumatic or indirect nerve damage (Bolton et al., 1998; Lim et al., 2014). Most studies on the functionality, cell biology and clinical significance of the interface between the circulating blood and the nervous system have been carried out by modeling the BBB rather than the BNB, but the apparent similarities may, at least to some extent, allow transfer of knowledge to BNB modeling. Key aspects to evaluate with a BNB model include: paracellular and transcellular trafficking, evaluation of BNB permeability to drugs and ultimately understanding of how to restore BNB permeability to its normal level. Adequate human cell lines derived from the BNB have until recently not been available limiting the number of BNB modeling studies. The key problem with *in vitro* cell lines is that they potentially neither retain physiological nor morphological properties. However, recently, a conditionally immortalized human peripheral nerve microvascular endothelial cell line and human pericyte cell lines have been developed (Sano and Kanda, 2011; Abe et al., 2012), which appear to retain the desired properties, such as expression of the key tight junction proteins claudin-5, occludin, ZO-1 and ZO-2 (Figure 1D) as well as key influx and efflux transporters (Abe et al., 2012), thereby increasing the toolbox for BNB modeling. Additional highly valuable information has recently been gathered by transcriptome sequencing of cultured primary endoneurial endothelial cells as well as by laser-capture microdissected endoneurial microvessels from human sural nerves (Palladino et al., 2017). The information regarding the molecular composition of the intact adult human BNB may form the basis for novel identifications of molecular changes in diabetes and DN that may be further investigated in *in vitro* BNB models.

## Perspectives in BNB Research

Selective pharmacological targeting of damaged nerve fibers without affecting healthy fibers is the outmost clinical goal. Accordingly, it has been shown in a rat model that it is possible to artificially open the BNB in a transient manner thereby enhancing the activity of pain relieving opioids at peripheral sites. In addition, perineurial injection of claudin-1 siRNA downregulated the TJ protein claudin-1, enabling an opioid peptide and a selective sodium channel to exert antinociceptive effects (Antonijevic et al., 1995; Hackel et al., 2012). Therefore, this drug delivery model may be applicable to various forms of neuropathic pain as well as to numerous neuroinflammatory and degenerative disorders in which neural barriers are disrupted (Lim et al., 2014). BNB endothelial cells are the only cells that come into direct contact with the blood constituents. Therefore, another approach may be to systemically manipulate this first-line barrier against transcellular entry of blood-borne pathogenic cells by targeting various endothelial adhesion molecules and chemokine receptors (mediating recruitment of circulating leukocytes into the PNS parenchyma). TJs constitute another obvious target and have been shown to be affected by the structural analog of sphingosine, fingolimod, which appears to enhance BNB barrier properties by upregulating the TJ protein claudin-5 upon interacting with endothelial cells (Nishihara et al., 2018). Fingolimod is used for treatment of demyelinating diseases like multiple sclerosis, where its therapeutic effect relies on decreasing egress of lymphocytes from lymph nodes. However, it has been suggested that part of its therapeutic effect might be due to its BBB enhancing properties (Nishihara et al., 2018). In line with this, fingolimod may prove beneficial in other demyelinating polyneuropathies and for restoration of the BNB in diabetic conditions. Yet other BNB elements, the BNB pericytes, may constitute a drug target. BNB pericytes produce various growth factors as well as several neurotrophic factors, including nerve growth factor (NGF), brain-derived neurotrophic factor (BDNF), and glial cell-derived neurotrophic factor (GDNF) (Shimizu et al., 2011a). GDNF secreted from peripheral nerve pericytes has been found to be one of the key molecules responsible for upregulation of claudin-5 expression in the BNB (Shimizu et al., 2012) indicating that regulation of trophic factor secretion from pericytes may modify BNB functions, potentially facilitating axonal regeneration and remyelination in DN.

Taken together, obtaining a better understanding of the pathological molecular mechanisms occurring during BNB cellular dysfunction in diabetes is key to identification of potential drug targets.

## Conclusion

Breakdown of the BNB is a largely unknown aspect of DN. Numerous studies show that blood-borne cells and soluble molecules play a role in development of DN by means of inducing aberrant BNB permeability, endoneurial hypoxia and degeneration of pericytes (summarizing Table 1). Together, this has reignited interest in microvascular disabilities as an important factor for neuropathy progression. Increasing evidence suggests that such abnormalities, however, not only occur at full throttle once DN has been established, but also contribute to its development, as some diabetic patients without neuropathy have been found to display increased BNB permeability. Improved mechanistic understanding of the BNB response to diabetes could unravel novel molecular therapeutic targets to ameliorate the disease and be key to fundamentally understand how neuropathies develop in some patients but not in others. Manipulation of such targets at onset or in the early stages of the disease may reduce the extent of demyelination and axonal damage, ultimately improving the prognosis for diabetic patients. Harnessing the early BNB breakdown in diabetic patients, an ideal future strategy for DN treatment would involve BNB-impermeable analgesic agents. These drugs would have the benefit of having selective access only to the sites where the BNB is disrupted, ideally affording selective localization of drugs to damaged neural tissues over healthy nerve fibers.

**Table 1:** Summarizing table with the main described mechanisms involved in DN that might contribute or be direcly linked with BNB breakdown.

Mechanisms involved in BNB breakdown	
Increased permeability	Rechthand et al., 1987; Poduslo et al., 1988; Cameron et al., 2001.
Inflammation	Grant, 2007; Nguyen et al., 2012; O’Brien et al., 2015; Zhu et al., 2015; Hinder et al., 2018.
Endoneurial hypoxia	Tuck et al., 1984; Newrick et al., 1986; Cameron et al., 1994; Ibrahim et al., 1999; Lim et al., 2015; Ostergaard et al., 2015; Kan et al., 2018.
Pericyte degeneration	Giannini and Dyck, 1995; Cameron et al., 2001; Shimizu et al., 2011b.


## Author Contributions

MR, NF, AD, TSJ, CBV, and NPG equally contributed for the writting of the manuscript and approved it for publication.

## Conflict of Interest Statement

The authors declare that the research was conducted in the absence of any commercial or financial relationships that could be construed as a potential conflict of interest.
